# Supplementary material for: Challenges and commonly used countermeasures in the implementation of lifelong antiretroviral therapy for PMTCT in Central Uganda: Health providers’ perspective
Source: PLoS One. 2023 Jan 20;18(1):e0280893. doi: 10.1371/journal.pone.0280893 (PMC9858842; doi:10.1371/journal.pone.0280893)
Supplement: Attachment — Submitted filename: Response to Reviewers.docx [file pone.0280893.s002.docx]

PONE-D-22-14861R1

Challenges and commonly used countermeasures in the implementation of lifelong antiretroviral therapy for PMTCT in Central Uganda: Health providers’ perspective

Dear Academic Editor,

Thank you so much for all the additional comments and suggestions that were raised during the review of the re-submitted manuscript. We thank the reviewers for all the comments. They have enabled us to improve our manuscript further.

We have addressed all comments in the “Point by point” response letter below. The changes are in track changes in the file labeled “Revised Manuscript with Track Changes”.

Please include the following items when submitting your revised manuscript:

A rebuttal letter that responds to each point raised by the academic editor and reviewer(s). You should upload this letter as a separate file labeled 'Response to Reviewers'.

A marked-up copy of your manuscript that highlights changes made to the original version. You should upload this as a separate file labeled 'Revised Manuscript with Track Changes'.

An unmarked version of your revised paper without tracked changes. You should upload this as a separate file labeled 'Manuscript'.

We have included all the above items.

If you would like to make changes to your financial disclosure, please include your updated statement in your cover letter. Guidelines for resubmitting your figure files are available below the reviewer comments at the end of this letter.

We have not made any changes to the financial disclosure.

Please review your reference list to ensure that it is complete and correct. If you have cited papers that have been retracted, please include the rationale for doing so in the manuscript text, or remove these references and replace them with relevant current references. Any changes to the reference list should be mentioned in the rebuttal letter that accompanies your revised manuscript. If you need to cite a retracted article, indicate the article’s retracted status in the References list and also include a citation and full reference for the retraction notice.

We have reviewed the reference list to ensure that it is complete and correct.

[Note: HTML markup is below. Please do not edit.]

Reviewers' comments:

Reviewer's Responses to Questions

Comments to the Author

1. If the authors have adequately addressed your comments raised in a previous round of review and you feel that this manuscript is now acceptable for publication, you may indicate that here to bypass the “Comments to the Author” section, enter your conflict of interest statement in the “Confidential to Editor” section, and submit your "Accept" recommendation.

Reviewer #1: All comments have been addressed

Reviewer #2: All comments have been addressed

______________________________________

2. Is the manuscript technically sound, and do the data support the conclusions?

The manuscript must describe a technically sound piece of scientific research with data that supports the conclusions. Experiments must have been conducted rigorously, with appropriate controls, replication, and sample sizes. The conclusions must be drawn appropriately based on the data presented.

Reviewer #1: Yes

Reviewer #2: Yes

_______________________________________

3. Has the statistical analysis been performed appropriately and rigorously?

Reviewer #1: N/A

Reviewer #2: N/A

________________________________________

4. Have the authors made all data underlying the findings in their manuscript fully available?

The PLOS Data policy requires authors to make all data underlying the findings described in their manuscript fully available without restriction, with rare exception (please refer to the Data Availability Statement in the manuscript PDF file). The data should be provided as part of the manuscript or its supporting information, or deposited to a public repository. For example, in addition to summary statistics, the data points behind means, medians and variance measures should be available. If there are restrictions on publicly sharing data—e.g. participant privacy or use of data from a third party—those must be specified.

Reviewer #1: No

Reviewer #2: No

________________________________________

5. Is the manuscript presented in an intelligible fashion and written in standard English?

PLOS ONE does not copyedit accepted manuscripts, so the language in submitted articles must be clear, correct, and unambiguous. Any typographical or grammatical errors should be corrected at revision, so please note any specific errors here.

Reviewer #1: Yes

Reviewer #2: Yes

________________________________________

6. Review Comments to the Author

Please use the space provided to explain your answers to the questions above. You may also include additional comments for the author, including concerns about dual publication, research ethics, or publication ethics. (Please upload your review as an attachment if it exceeds 20,000 characters)

Reviewer #1: Authors response adequately addresses the comments raised. The manuscript is much clearer. However, the authors need to correct the error regarding Reference 2 and 3 Otherwise, the manuscript is good to go

Thank you. We have corrected the error in the references. They are now referenced 2 (Lines 771-772) and 4 (Lines 778-779).

Reviewer #2: Thank you for addressing the review comments. I only have a few minor additional comments.

Please do a final copy edit before finalising.

Thank you. We have edited the final copy of the manuscript.

Introduction:

Lines 87-91: Revision describing that MTCT of HIV should have declined due to revised treatment guidelines – the example demonstrating that it has not declined, uses data from 2017-2019, yet the guidelines were only implemented in 2020 (Line 78). Perhaps authors should find more recent data on MTCT rate in Uganda, or revise the wording of the paragraph.

We have revised the wording and included more recent data as well (Lines 95-102).

Methods:

Study population: The study population selection and participation is much clearer now, thank you.

Thank you very much.

Results:

Line 229: Insert missing words: “Table 2 shows the details of the health providers who participated…”

We have revised the wording of the statements (Lines 221 and 227).

________________________________________

7. PLOS authors have the option to publish the peer review history of their article (what does this mean?). If published, this will include your full peer review and any attached files.

If you choose “no”, your identity will remain anonymous but your review may still be made public.

Do you want your identity to be public for this peer review? For information about this choice, including consent withdrawal, please see our Privacy Policy.

Reviewer #1: No

Reviewer #2: No

________________________________________

[NOTE: If reviewer comments were submitted as an attachment file, they will be attached to this email and accessible via the submission site. Please log into your account, locate the manuscript record, and check for the action link "View Attachments". If this link does not appear, there are no attachment files.]

While revising your submission, please upload your figure files to the Preflight Analysis and Conversion Engine (PACE) digital diagnostic tool, https://pacev2.apexcovantage.com/. PACE helps ensure that figures meet PLOS requirements. To use PACE, you must first register as a user. Registration is free. Then, login and navigate to the UPLOAD tab, where you will find detailed instructions on how to use the tool. If you encounter any issues or have any questions when using PACE, please email PLOS at figures@plos.org. Please note that Supporting Information files do not need this step.
